# EVALUATING AND ADDRESSING ASPECTS OF SOCIAL HEALTH AMONG AGING POPULATIONS

**DOI:** 10.1093/geroni/igad104.1391

**Published:** 2023-12-21

**Authors:** Allison Marziliano, Thomas Cudjoe

## Abstract

The physical and psychological aspects of human functioning have traditionally been at the heart of gerontological research. In recent years, however, social health, including such concepts as social isolation, social support and loneliness, have received increasing attention. This symposium describes research in this developing area, starting with a description of the evaluation of disparities in social health and culminating with an overview of the development process for interventions to improve poor social health. First, Victor and Taylor, in their respective presentations, focus on identifying disparities (racial, ethnic, gender, marital status, household composition) in social health across older adults in two geographic regions (United Kingdom, United States). Next, Marziliano describes the iterative process of developing 6 brief (~5 minute) videos to administer an adaptation of Meaning-Centered Psychotherapy for the purpose of increasing meaning and reducing loneliness in caregivers of persons with Alzheimer’s Disease (AD) or AD-related dementias (ADRD). Development of this intervention consisted of three phases: preparatory work among the study team, interviews to collect stakeholder feedback with 15 AD caregivers, and construction of the web-based tool. Lastly, guided by Intervention Mapping, the Health Belief Model and Social Cognitive Theory, Thomas describes the process of developing an educational video and website to address social isolation among homebound older adults. This process incorporated multiple steps: a needs assessment during two focus groups; literature review; pilot of educational materials; and finally, implementation and evaluation of the intervention.

